# Antioxidant and anticancer activities of *Trigonella foenum-graecum, Cassia acutifolia and Rhazya stricta*

**DOI:** 10.1186/s12906-018-2285-7

**Published:** 2018-08-22

**Authors:** Bayan Al-Dabbagh, Ismail A. Elhaty, Ala’a Al Hrout, Reem Al Sakkaf, Raafat El-Awady, S. Salman Ashraf, Amr Amin

**Affiliations:** 1 Department of Chemistry, College of Science, UAE University, PO Box 15551, Al Ain, UAE; 2 Department of Biology, College of Science, UAE University, PO Box 15551, Al Ain, UAE; 3 Zoology Department, Cairo University, Giza, Egypt; 4 Department of Pharmacy Practice and Pharmacotherapeutics, Sharjah Institute for Medical Research and College of Pharmacy, University of Sharjah, Sharjah, UAE

**Keywords:** Antioxidants, Anticancer, Medicinal plants, Traditional medicine, *Rhazya stricta*

## Abstract

**Background:**

Here, we determined in vitro antioxidant activity, total phenols and flavonoids and evaluated antiproliferative activity of three medicinal plant extracts: *Trigonella foenum-graecum* (Fenugreek), *Cassia acutifolia* (Senna) and *Rhazya stricta* (Harmal).

**Methods:**

The leaves of the three medicinal plants were extracted with 70% ethanol. Antioxidant activities of the extracts were determined by using DPPH (1,1-diphenyl-2-picrylhydrazyl) assay. Total flavonoid and phenolic contents were determined using colorimetric assays. MTT assay was used to estimate the antiproliferative activities of the extracts against human hepatoma (HepG2) cancer cell line. In addition, the effects of *R. stricta* extract on cell cycle, colony formation, and wound healing of HepG2 cells and tube formation of HUVEC cells were assessed.

**Results:**

Percentage inhibition of DPPH scavenging activity were dose-dependent and ranged between (89.9% ± 0.51) and (28.6% ± 2.07). Phenolic contents ranged between (11.5 ± 0.013) and (9.7 ± 0.008) mg GAE/g while flavonoid content ranged between (20.8 ± 0.40) and (0.12 ± 0.0.01) mg QE/g. Antiproliferative results of the extracts were found to be consistent with their antioxidant activity. Among the extracts evaluated, that of *R. stricta* showed the best antioxidant, antiproliferative and antimetastatic activities at low concentration. It also inhibited the colony-formation capacity of HepG2 cells and exhibited antiangiogenic activity. Cell cycle analysis showed significant arrest of cells at G2/M phase 12 and 48 h after treatment and significant arrest at G1/S phase after 24 h of treatment. Consistent data were observed in western blot analysis of protein levels of Cdc2 and its cyclin partners.

**Conclusions:**

These findings introduce *R. stricta* as a potentially useful anti-metastatic agent and a novel potential anti-tumour agent for hepatocellular carcinoma (HCC) treatment.

**Electronic supplementary material:**

The online version of this article (10.1186/s12906-018-2285-7) contains supplementary material, which is available to authorized users.

## Background

Free radicals are mainly produced by oxidation processes and they have an important role in the processes of food spoilage and chemical materials degradation. They also contribute to human disorders such as aging-associated diseases, cardiovascular diseases, cancer and inflammatory diseases [1, 2]. Free radicals may also cause a depletion of the immune system antioxidants, a change in the gene expression and may induce the synthesis of abnormal proteins. About 5% or more of the inhaled oxygen (O_2_) is converted to reactive oxygen species (ROS) such as O_2_^−^, H_2_O_2_, and OH radicals. ROS represents the major type of free radicals in any biological system. They are produced through the mitochondrial electrons transport chain [2, 3].

Antioxidants are used to neutralize the effects of free radicals. Thus, they protect humans against infection and degenerative diseases. Antioxidants can be classified into two major categories, natural and synthetic. Synthetic antioxidants include butylated hydroxy anisole (BHA), butylated hydroxyl toluenes (BHT), tertiary butylated hydroquinone and gallic acid esters. These antioxidants effectively inhibit oxidation, may serve as chelating agents such as ethylene diamine tetra acetic acid (EDTA), and can bind metals reducing their contribution to the process [4]. However, antioxidants are thought to cause or promote negative health effects such as mutagenesis and carcinogenesis in humans [5]. Therefore, there is a strong trend to replace the synthetic with naturally occurring antioxidants that can prevent free radical-related diseases [4, 6].

Natural antioxidants help in controlling the formation of free radicals and activated oxygen species or they can inhibit their reaction with biological structures [7]. These antioxidants include antioxidative enzymes, such as superoxide dismutase, catalase, and glutathione peroxidase, and small nonenzymatic antioxidant molecules, such as glutathione and vitamins C and E [8]. Many herbs and spices (rosemary, thyme, oregano, sage, basil, pepper, clove, cinnamon, nutmeg, and saffron), and plant extracts (tea, grapeseed, and lemon balm) contain antioxidant components [9–11].

In this study, three hydroalcoholic extracts of traditionally medicinal plants used in the United Arab Emirates (UAE) were evaluated for their antioxidant activities, phenol and flavonoid contents. These plants are, *Trigonella foenum-graecum*, locally known as “Helba”, *Cassia acutifolia*, commonly known as “Holoul” and *Rhazya stricta* which is locally called “Harmal”.

*T. foenum-graecum* seeds are traditionally used as herbal medicine for their carminative, tonic, aphrodisiac and anticancer effects [12–14]. The leaves of *C. acutifolia* is frequently used in folk medicine as a purgative for a long time [15]. The extracts of *R. stricta* leaves are traditionally used for the treatment of various disorders such as diabetes, sore throat, helminthiasis, inflammatory conditions and rheumatism [16].

Available treatment for hepatocellular carcinoma are mainly limited to invasive hepatectomy or chemotherapy. However, the attention has shifted in recent years to natural-based products for candidate anticancer therapeutics. In the present study, the antiproliferative effects of *Trigonella foenum-graecum*, *Cassia acutifolia*, and *Rhazya stricta* on hepatoma cell line HepG2 were investigated. The use of HepG2 cells to test the cytotoxic effects of a wide range of drugs has been well documented, due to their wide availability, well-differentiation, and drug metabolizing activity [17].

Despite playing a key role in cellular processes, free radicals pose a threat to cells by damaging DNA, proteins, and cellular membranes, leading to onset of many diseases including cancer [18, 19]. Thus, by decreasing free radicals and oxidative stress, antioxidants play a role in ameliorating DNA damage, reducing the rate of abnormal cell division, and decreasing mutagenesis [20]. Therefore, many antioxidant-rich plants possess anticancer activity [21–23].

Vascular endothelial growth factor (VEGF) has been recognized to be involved in several stages of angiogenesis in malignant diseases by its multi-functional effects in activating and integrating signalling pathway networks [24]. VEGF signalling blockade reduces new vessel growth and leads to endothelial cell apoptosis. Therefore, using tyrosine kinase inhibitors or VEGF/VEGF receptor (VEGFR) antibodies to inhibit crucial angiogenic steps is a practical therapeutic strategy when treating neovascularisation diseases [25]. A potent angiogenesis inhibitor known as E7820, has been shown to reduce integrin α 2 mRNA expression and inhibit basic fibroblast growth factor/VEGF-induced HUVEC proliferation and tube formation [26, 27]. Integrin α 2β 1/α 1β 1 expression is reportedly regulated by VEGF and an inhibitory antibody against α 2β 1/α 1β 1 has been shown to inhibit angiogenesis and tumour growth in VEGF-overexpressing tumour cells [28, 29]. Therefore, we investigated here the effect of *R. stricta* leaves extract on angiogenesis utilizing HUVEC tube formation assay; as it showed the most promising antiproliferative activity. In addition, this work was set to determine the in vitro antioxidant activity, total phenols and flavonoids, anticancer activities of tested plants with special interest in *R. stricta*.

## Methods

### Chemicals

All solvents were analytical grade. Agilent Cary 60 UV-Vis Spectrophotometer was used in all spectrophotometric measurements. Ascorbic acid, ferric chloride, aluminium chloride, potassium acetate, quercetin, DPPH reagent, Folin-Ciocalteau reagent, gallic acid, sodium carbonate, methanol and ethanol were obtained from Sigma Chemical Co. (St. Louis, MO, USA). Millipore deionized water was used throughout. Thiazolyl Blue Tetrazolium Bromide (Sigma Aldrich, USA), Dimethyl Sulfoxide (Sigma Aldrich, USA).

### Plant samples

Dried leaves of *C. acutifolia*, *R. stricta* and *T. foenum-graecum* were purchased from the local market. The taxonomic authentication of all the plants was carried out by Dr. Fatima Al-Ansari at the Biology Department, College of Science, United Arab Emirates University. Voucher specimens were deposited at the herbarium of the Biology Department (voucher reference numbers: BA2018–1, BA2018–2, BA2018–3).

### Preparation of plant extracts

The leaves of the medicinal plants were crushed separately in a grinder. A sample of 10 g of each plant was extracted with 150 mL of 70% ethanol and 30% water as it showed the best extraction yield [30]. The crushed plants were macerated for 48 h at 4 °C. The resulting mixture was then filtered under vacuum and concentrated under reduced pressure in a rotary evaporator at 40 °C. The extracts were further dried using a TELSTAR CRYODOS freeze dryer machine then kept at − 20 °C for further analysis. A solution of 30 mg/mL of each plant was prepared in 50% ethanol for the following tests.

### Determination of total polyphenol content

The total phenolic content (TPC) was determined by using the Folin-Ciocalteau reagent [31]. A 10% solution was prepared from the stock solution (30 mg/mL) using 50% ethanol. 100 μl of this solution was mixed with 200 μl of the Folin-Ciocalteau reagent and 2 mL of de-ionized water then incubated at room temperature for 3 min. A sample of 20% aqueous sodium carbonate (*w*/w, 1 mL) was then added to the mixture. The total polyphenols were determined after 1 h of incubation at room temperature. A negative control sample was also prepared using the same procedure. The absorbance of the resulting blue colour was measured at 765 nm. Results were expressed in mg gallic acid equivalents (GAE) per g dry weight of plant material using an equation obtained from gallic acid calibration curve. The samples were analyzed in triplicate.

### Free-radical scavenging activity

The antioxidant activity of the extracts was assessed based on their ability to scavenge the stable 1,1-diphenyl-2-picrylhydrazyl (DPPH) radical as described previously [32]. Various concentrations of the three extracts in methanol were prepared (0.15 to 1.5 mg/mL). A methanolic solution of DPPH (3.8 mL, 60 μg/mL) was rapidly mixed with the plant extract (200 μl, 30 mg/mL) in a test tube, with methanol serving as the blank sample and a control was also assayed simultaneously. The contents of the tubes were swirled then allowed to stand for 30 min at room temperature in the dark. The absorbance was measured at 517 nm in a spectrophotometer. The scavenging ability of the plant extract was calculated using this equation: DPPH Scavenging activity (%) = [(Abs control–Abs sample)]/ (Abs control)] × 100, where Abs control is the absorbance of DPPH + methanol; Abs sample is the absorbance of DPPH radical + sample (sample or standard). The EC_50_ value (μg/mL), the effective concentration at which DPPH· radicals are scavenged by 50%, was determined graphically. The total antioxidant activity was expressed as ascorbic acid equivalent/g dry extract. The assay was done in triplicates.

### Determination of total flavonoids

The total flavonoids content in the extracts was determined using the aluminium chloride colorimetric method [33]. A known concentration (600 μg/mL) of each extract in methanol was prepared. A 500 μl of the extracts were mixed separately with 0.1 mL of 10% (*w*/*v*) aluminium chloride solution, 0.1 mL of 1 M potassium acetate solution, 1.5 mL of methanol and 2.8 mL of distilled water. The solutions were thoroughly mixed and incubated at room temperature for 30 min. The absorbance of the reaction mixture was measured at 415 nm using a spectrophotometer. The total flavonoids content was determined using a standard curve with quercetin (1 to 25 μg/mL) as the standard. The mean of three readings was used and expressed as mg of quercetin equivalents (QE)/ g of the dry extract.

### Cell culture

A human hepatocellular carcinoma (HCC)-derived cell line (HepG2) was cultured in RPMI 1640 medium containing 1% antibiotic cocktail and supplemented with 10% fetal bovine serum. Cells were incubated at 37 °C in 5% CO_2_ humidified incubator. Cells were passaged every 2–3 days using 0.25% trypsin-EDTA.

### Cytotoxicity assay

HepG2 were seeded at a density of 5000 cells/well in a 96-well plate, and were allowed to attach overnight. Thereafter, cells were treated with various concentrations of the plants extracts for 24 h. To assess the cytotoxic effect of the three plants extracts, MTT (3-[4,5-dimethylthiazol-2-yl]-2,5-diphenyltratrazolium bromide) assay was carried out. Briefly, cells treated with the plant extracts were exposed to tetrazolium MTT at a concentration of 5 mg/mL. Viable active cells reduced yellow MTT salt to insoluble purple formazan, which was dissolved using DMSO. The absorbance of the coloured solution was measured at a wavelength of 570 nm using Epoch microplate spectrophotometer (BioTek). The obtained absorbance at 570 nm of both control and treated cells was used to calculate percentage of cell viability. Assuming 100% viability in control cells, percentage of treated cells viability will be calculated accordingly:

$$ \mathrm{Percent}\ \mathrm{of}\ \mathrm{viable}\ \mathrm{cells}=\left(\mathrm{Abs}.\mathrm{of}\ \mathrm{treated}\ \mathrm{cells}/\mathrm{Abs}.\mathrm{of}\ \mathrm{control}\ \mathrm{cells}\right)\times 100 $$

### Assessment of morphological changes

HepG2 were seeded at a density of 0.25 × 10^6^ cells/ well in a 6-well plate, and were allowed to attach overnight. After which, cells were treated without (0 μg, control) or with increasing concentrations of *R. stricta* (10, 20, 30, 50, 70 μg) for 24 h. The morphology of the cells was assessed after being fixed and stained with 0.5% crystal violet using bright-field microscopy (200 x magnification, scale = 200 μm).

### Colony formation assay

To assess the effects of *R. stricta* on cell survival, the colony formation assay was carried out in vitro. Briefly, HepG2 cells were seeded at a density of 1000 cells/ well in a 6-well plate, and were incubate for 24 h to allow attachment. The second day, the cells were treated without (0 μg, control) or with increasing concentrations (10, 20, 30 μg) of the extract for 24 h. After which, the media was replaced with fresh complete growth media without the extract, and cells were left to incubate until visible colonies were formed; while changing the media every 3–4 days. The experiment was carried out in triplicates. Colonies were fixed with absolute methanol, then stained with 0.5% crystal violet. Results are represented as the percentage of the well area that is covered by colonies (colony area percentage). Analysis has been carried out using ImageJ plugin ColonyArea [34]. In addition, an absorption-based method was carried out to validate the earlier results, by which the absorption of the crystal violet dye in each well is measured after being dissolved. Briefly, the samples that had been analysed using ImageJ were subjected to 10% acetic acid solution, then were placed on an orbital shaker for 15 min. After which, 100 μL of each triplicate sample was transferred to a 96-well plate (in triplicates), and absorbance was measured using Epoch microplate spectrophotometer (BioTek).

### Wound-healing assay

To assess the ability of HepG2 to migrate after the treatment with *R. stricta*, wound-healing assay was carried out in vitro. Cells were seeded at a density of 0.5 × 10^6^/ well in 6-well plate, and were allowed to attach overnight. A scratch in the cell monolayer was made using a sterile plastic pipetting tip, and then the monolayer was washed with PBS. The cells were treated without (0 μg) or with 20, 30 μg of the extract. Images were taken at 0, 24, 48, 72 h using bright-field microscopy (40 x magnification). Analysis was carried out using ImageJ, percent of open area was calculated according to the following formula [T_x_ = T_24,_ T_48,_ or T_72_ (at time 24, 48, or 72 h, respectively]: Percent of open area = (open area at T_x_/ open area at T_0_) X 100.

Experiment was carried out in triplicates, data is representative of 3 random regions in each triplicate of each sample.

### Cell cycle analysis

Effect of *R. stricta* extract on cell cycle progression of HepG2 cells was analysed as previously described [35]. Cells were treated without or with 30 μg of *R. stricta* extract at different time intervals (6 – 48 h), collected by trypsinization, washed twice with PBS, fixed in 70% ethanol, treated with RNase, stained with propidium iodide and then cell cycle distribution was analysed in BD Accuri C6 cytometer and software (BD Biosciences, USA).

### Western blotting

HepG2 were seeded at a density of 1 × 10^6^ in 60 mm dish and allowed to attach overnight. Cells were treated without or with 30 μg of extract for 6, 12, 24, 48 h. Cells were lysed and total protein was quantified using BCA. 20 μg of total protein was separated on SDS-PAGE, transferred onto nitrocellulose membranes that were blocked using 5% BSA TBST. Primary antibodies against Cdc2 (1:000; cell signalling), p-Cdc2 (1:1000; cell signalling), Cyclin B1 (1:1000; cell signalling), Cyclin A1 (1:000; Abcam) were used. GAPDH (1:15000; Abcam) was used as loading control. Proteins were detected using LI-COR C-DiGit Chemiluminescence Western Blot Scanner.

### Matrigel capillary tube formation

96-well plate was coated with Matrigel matrix (Corning, NY, USA) at 50 ul/well and allowed to polymerize for 60 min at 37 °C. HUVEC cells were then seeded on the Matrigel at a concentration of 2 × 10^4^ cells/well without (0 μg) or with (10, 20, 30 μg) *R. stricta* extract. After incubation for 18 h, tubules were imaged using an inverted microscope and analyzed with ImageJ software.

### Statistical analysis

All data were expressed as mean ± standard deviation (SD) of three independent experiments. Correlation analysis of antioxidants versus the total phenolic and flavonoid contents were carried out using the regression analysis, with GraphPad Prism 6.0 and Microsoft Excel 2016. *P* < 0.05 was considered to indicate a significant difference.

## Results and discussion

Medicinal plants have been of great interest as a source of natural antioxidants used for health promotion. The therapeutic activity of plants is mostly due to their biologically active polyphenolic substances, mostly flavonoids and phenolic acids. These substances exhibit antioxidant, anti-lipoxygenase and anticancer activities. The present study elaborates on the antioxidant activity, polyphenolic and flavonoid contents of three folk plants from the UAE; *T. foenum-graecum*, *C. acutifolia* and *R. stricta.* The antiprolifrative effect of such plants was studied against human cancer cells HepG2 in an attempt to find a correlation with the antioxidant activity of those extracts that are based on their phenolic and flavonoid contents.

### Plant extraction

Different solvents have been used in the literature for the preparation of plant extracts [36]**.** In this study, we used 70% ethanol as the extraction solvent. The amorphous solid of the leave extracts under investigation was obtained by complete evaporation of ethanol/water. The yield of each extract was calculated as *w*/w percent yield. The yields of *T. foenum-graecum*, *C. acutifolia* and *R. stricta* extracts were 25, 23 and 30% respectively.

### Total polyphenol content of the extracts

**Fig. 1:**
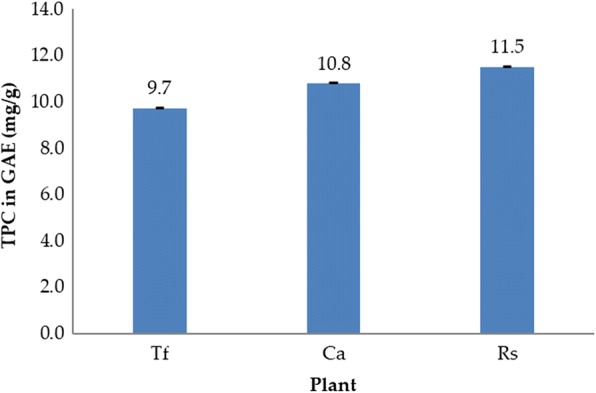
Total phenolic content of *T. foenum-graecum* (Tf), *C. acutifolia* (Ca) and *R. stricta* (Rs) extracts determined by the Folin-Ciocalteau assay and calculated as mg GAE/g extract based on dry weight. Results are the average of triplicates ± SD

Polyphenols are aromatic secondary plant metabolites and are widely spread throughout plants. They have been associated with colour, sensory qualities, and nutritional and antioxidant properties of food [37]. It is reported that there is a strong relationship between total polyphenol contents and antioxidant activity. The hydroxyl groups in phenols have a strong scavenging ability for free radicals. Therefore, the total polyphenol contents of plants may directly contribute to their antioxidant activity [32, 38]. The Folin-Ciocalteau reagent is commonly used in the literature to determine phenolic compounds. This reagent reacts with phenolic compounds and gives a blue colour complex that absorbs radiation and allows quantification [39]. The total phenolic content for the ethanolic extracts of *C. acutifolia*, *R. stricta* and *T. foenum-graecum* was determined by the Folin-Ciocalteau method using gallic acid as a standard. The calibration curve showed linearity for gallic acid in the range of 0.5–26 μg/mL, with a correlation coefficient (R^2^) of 0.984. *R. stricta* contained the highest total polyphenols (11.5 ± 0.013 mg GAE/g extract), followed by *C. acutifolia* (10.8 ± 0.025 mg GAE/g extract) and *T. foenum-graecum* (9.7 ± 0.008 mg GAE/g extract) (Fig. 1). Belguith-Hadriche et al. [40] reported the total phenolic content of various extracts of *T. foenum-graecum* ranged between 9.42 ± 0.50 in hexane and 78.1 ± 0.90 mg GAE/g dry weight extracts in methanol. A study conducted in Saudi Arabia showed the total phenolic content of *R. stricta* extracts ranged between 62.5 ± 0.2 and 66.63 ± 0.03 mg GAE/g extract [41].

### The DPPH radical scavenging activity

The antioxidant activity of each plant was also assessed based on its ability to reduce the stable DPPH radical according to the method reported by Lim [32]. The DPPH radical (DPPH^•^) is a stable radical and has the ability to accept an electron or hydrogen radical and form a stable diamagnetic molecule producing a colour change from blue to yellow [42]. The colour change of DPPH has been widely used to measure the radical scavenging activity because of its stability, simplicity, and reproducibility [43]. The free radical scavenging capacity of the ethanolic extracts of the three plants were assayed based on the remaining amount (%) of DPPH^•^ as a function of time (30 min). The total antioxidant activity was expressed as ascorbic acid equivalent/g dry extract.

**Fig. 2:**
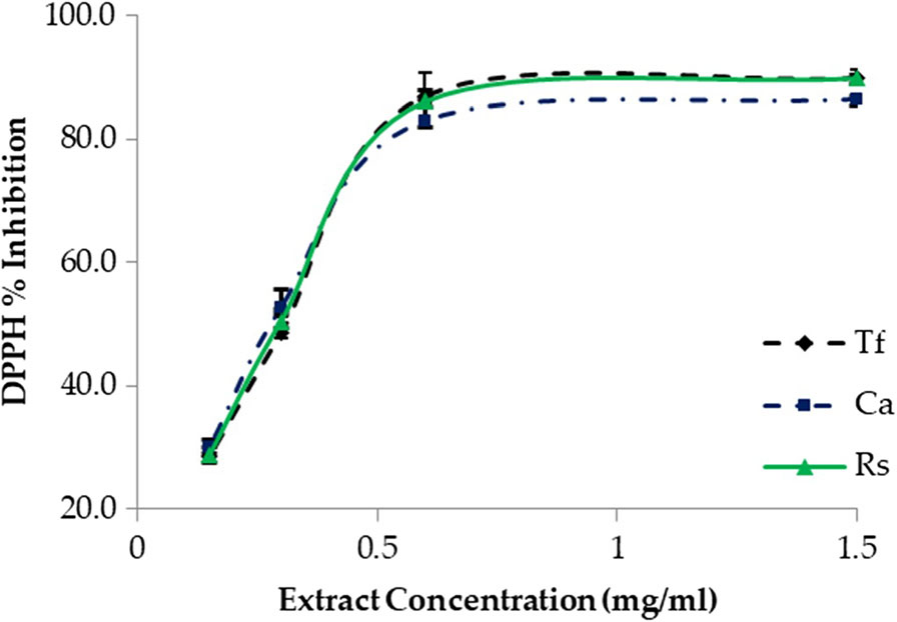
DPPH radical scavenging activities of the tested extracts

The calibration curve of ascorbic acid showed linearity in the range of 5–20 μg/mL, with a correlation coefficient (R^2^) of 0.994. The percentage inhibitions of DPPH scavenging activity in all the extracts were dose-dependent (Fig. 2). The DPPH scavenging activity by the *T. foenum-graecum* extract was (89.7% ± 1.54) at 1.5 mg/mL and (28.6% ± 2.07) at 0.15 mg/mL. DPPH of *C. acutifolia* was (86.3% ± 0.64) at 1.5 mg/mL and (30.0% ± 1.37) at 0.15 mg/mL while that of *R. stricta* was (89.9% ± 0.51) at 1.5 mg/mL and (28.7% ± 1.27) at 0.15 mg/mL. The results showed that there is no clear difference between the obtained DPPH scavenging activities of the three extracts. These results are almost in agreement with their total polyphenol contents. Consequently, the antioxidant activity of these plants might be related to their contents of phenolic compounds.

**Fig. 3:**
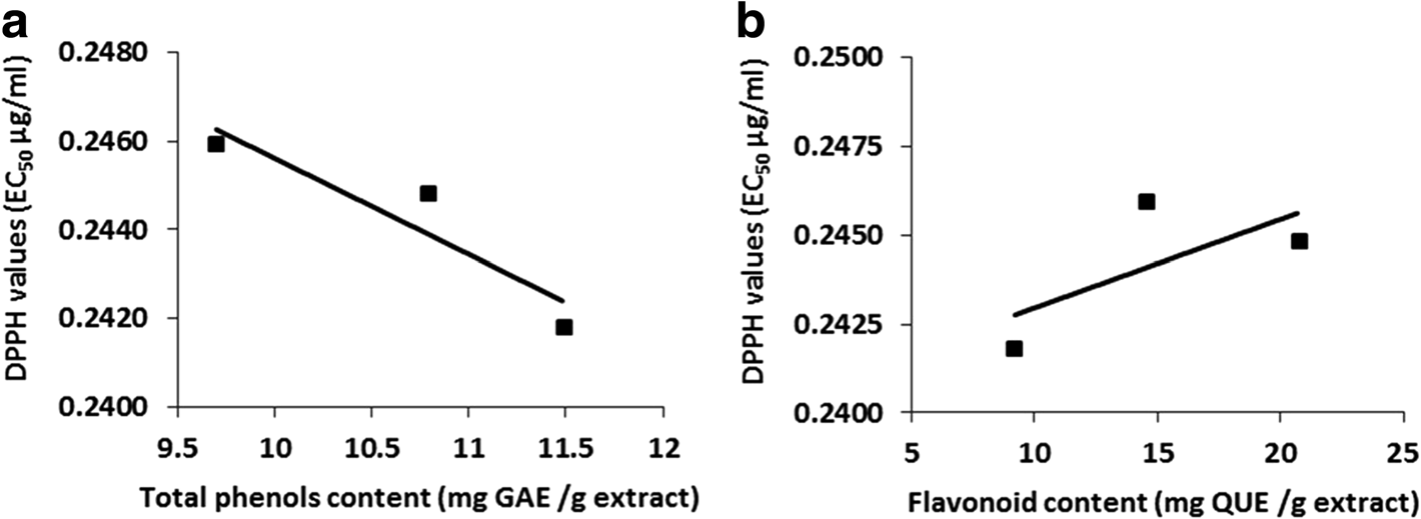
Linear correlations between the amount of total phenols and DPPH· radical scavenging activity (**a**) and between the flavonoid content and DPPH· radical scavenging activity (**b**)

In addition, the DPPH radical scavenging ability of the extracts was evaluated as EC_50_ (μg/mL) value. The smallest EC_50_ values indicates the best free radical scavenging activity. The highest scavenging activity was exerted by *R. stricta* (EC_50_ = 241.8 μg/mL) which contained the highest amount of total polyphenol content (11.5 μg/mL), followed by *C. acutifolia* (EC_50_ = 244.8, TPC = 10.8 μg/mL). The lowest radical scavenging activity was exhibited by the *T. foenum-graecum* extract (EC_50_ = 245.9, TPC = 9.7 μg/mL). The relationship between total phenol content and free radical scavenging activity (using EC_50_) was also studied using linear regression analysis (Fig. 3a). The results showed a significant negative correlation (R^2^ = − 0.856, *P*-value < 0.05) between EC_50_ (DPPH· scavenging) and total phenolic content suggesting that the presence of the phenolic compounds contributed significantly to the antioxidant activity of the tested plants. These results are consistent with previous works that showed a liner correlation between the total phenolic content and the reducing antioxidant capacity of some plant extracts [32, 38, 44].

### Total flavonoid contents

Flavonoids are a class of secondary plant phenolics. Flavonoids and their derivatives have a wide range of biological actions including anticancer activity. The anticancer activity of flavonoids is attributed to their potent antioxidant effects which include metal chelation and free-radical scavenging activities [45]. Flavonoids present in herbs were found to significantly contribute to their antioxidant properties [44]. The flavonoid content was obtained using aluminium chloride assay which based on the formation of a complex between the aluminium ion, Al (III), and the carbonyl and hydroxyl groups of flavones and flavonols that produce a yellow colour [46]. Flavonoid content was calculated from the regression equation of quercetin calibration curve and was expressed as quercetin equivalents. The calibration curve showed linearity in the range of 1–25 μg/mL, with a correlation coefficient (R^2^) of 0.999.

**Fig. 4:**
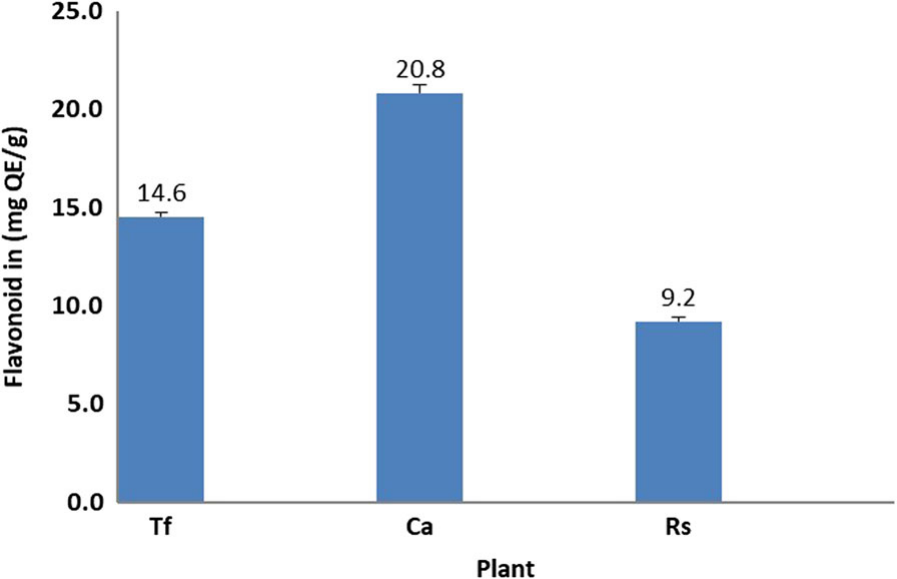
Flavonoids content of the tested extracts

Figure 4 shows the flavonoids contents in all the extracts. Using the standard curve generated by quercetin, the total flavonoids content of the *T. foenum-graecum* extract was (14.6 ± 0.21 mg QE/g), whereas, the *C. actutifolia* extract was (20.8 ± 0.40 mg QE/g) and finally that of *R. stricta* was (9.2 ± 0.22 mg QE/g).

The relationship between the total flavonoids content and the free radical scavenging activity (using EC_50_) was studied using linear regression analysis (Fig. 3b). The results showed a positive correlation (*R*^2^ = 0.460, *P*-value < 0.05) between EC_50_ (DPPH scavenging) and total flavonoids content. The obtained correlation was moderate suggesting that other compounds maybe participating in the radical scavenging activity of these plant extracts.

### Effects of extracts on cell viability in HepG2 cells

HCC remains among the leading cause of cancer-related death worldwide [47, 48]. Although therapeutic approaches for advanced HCC are limited to the use of multikinase inhibitors, such as sorafenib, only modest survival benefits have clinically been reported. Thus, identifying new compounds with promise antitumor activity against HCC is exceedingly needed [48, 49]. Natural products, especially from plants, are often better tolerated than their synthetic analogs used in cancer treatments [50]. They contain a wide spectrum of bioactive secondary metabolites that are the foundation of the recently introduced notion of broad-spectrum integrative approach for cancer prevention and treatment [51].

**Fig. 5:**
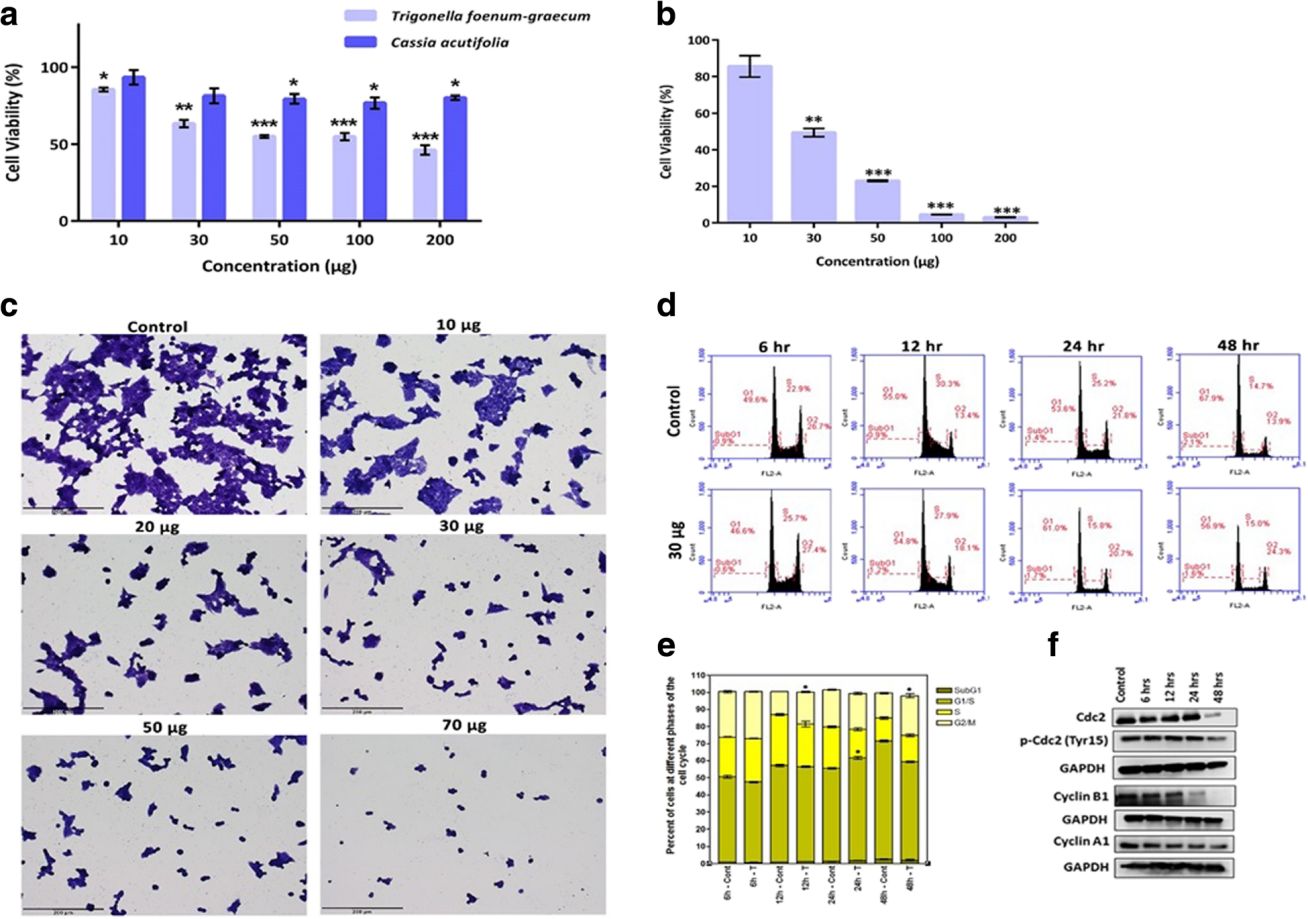
Assessment of the cytotoxic effects of *Trigonella foenum-graecum* (Helba), *Cassia acutifolia* (Holoul), and *Rhazya stricta* (Harmal) extracts on HepG2 in vitro. **a** MTT assay results of HepG2 cells viability after treatment with increasing concentrations of Helba and Holoul for 24 h. *P < 0.05, **P < 0.001, ***P < 0.0001 **b** MTT assay results of HepG2 cells viability after treatment with increasing concentrations of *R. stricta* extract for 24 h. *P < 0.05, **P < 0.001, ***P < 0.0001 **c** Assessment of morphological changes of HepG2 cells after treatment with increasing concentrations of *R. stricta* extract for 24 h. Cells were fixed and stained with crystal violet (scale bar = 200 μm). **d** Cell cycle progression of HepG2 cells after treatment with *R. stricta* extract at a dose of 30 μg over a period of 48 h. **e** Quantitative distribution of HepG2 cells in different phases of the cell cycle at different time intervals (*P < 0.05) **f** Immunoblot analysis of cell cycle regulatory proteins in HepG2 cells after treatment with *R. stricta* extract at a dose of 30 μg over a period of 48 h

Effects of tested extracts were investigated against human hepatoma (HepG2) cancer cell line. A dose-dependent reduction in cell viability was reported in cells treated with all tested extracts (Fig. 5). The IC_50_ values of extracts ranged from 30 μg/mL to 200 μg/mL. Treatment with *R. stricta* significantly enhanced the mortality of cancer cells at the lowest concentration (30 μg/mL). *T. foenum-graecum* was however less potent with IC_50_ of 200 μg/mL where 50% of HepG2 cancer cells were eradicated at 200 μg/mL. A fenugreek-enriched diet decreased colon tumour incidence and hepatic lipid peroxidation in liver cancer-induced rats in addition to increasing the endogenous antioxidant activities in liver [52]. Li et al. [53] showed that diosgenin, fenugreek’s main active ingredient, down regulated the expression of various STAT3-regulated genes, inhibited proliferation and potentiated the apoptotic effects of paclitaxel and doxorubicin, suggesting that diosgenin could be a novel and potential treatment option for HCC and other cancers. Therefore, the role of fenugreek extract and its active principals as supplements in diet-based preventive/therapeutic strategies to improve health care continues to be a fast growing field of research. The correlation between the antioxidant activities (free radical scavenging activity) and the anticancer activities (cell viability) of *C. acutifolia*, *R. stricta* and *T. foenum-graecum* extracts was studied using linear regression analysis. The results showed a significant positive correlation (*R*^2^ = − 0.933, 0.997 and 0.797, *P*-value < 0.05) for *C. acutifolia*, *R. stricta* and *T. foenum-graecum* extracts respectively.

At a concentration range of (100–200 μg/mL) *Cassia acutifolia’s* extract was similarly cytotoxic to HepG2 cells. Alkaloids extracted from Senna species reduced cell viability in a concentration-dependent manner of different tumour cell lines including HepG2 [49]. Senna alkaloids showed important antiproliferative activity on HepG2 cells that was mediated by ERK inactivation and down-regulation of cyclin D1 expression. Similarly, extracts of different cassia species were able to inhibit growth of colorectal (DLD1), among other, human cancer cell lines [54]. Thus, Senna’s extract may represent a potential new antitumor and/or adjuvant treatment against liver and colorectal cancer and further investigations should be conducted to unravel its molecular mechanism. *R. stricta*’s extract was by far the most effective cytotoxic agent tested at the present study at as little as 30 μg/mL. The δ-tocopherol and alkaloid fraction of *R. stricta*’s leaves extract have been shown to delay many angiogenic and inflammatory activities and to inhibit cell viability of HepG2, hence potentially useful against cancer [55, 56]. As *R. stricta* extract showed the most promising antiproliferative activity, its antiproliferative effects were tested against other GI cell line. The present study also showed that *R. stricta* extract inhibited cell proliferation in colorectal cancer cells (HCT116), in a dose -dependent manner (Additional file 1: Figure S1). To get insight into the molecular effects of *R. stricta* on HepG2 cells, its effect on cell cycle progression was analysed. *R. stricta* treatment induced arrest of cells in the G2/M phase (12 and 48 h time points) and in G1/S phase (24 h time point) (Fig. 5d and e). This cell cycle arrest reflects the cytotoxic effects of *R. stricta* on HepG2 cells where the cells are arrested at these phases to repair the toxic lesions induced by *R. stricta* or to be removed by death pathways such as apoptosis. The significant increase of the fraction of cells in G2/M after long term treatment (48 h) may indicate permanent arrest of cells in this phase (quiescence). These findings are consistent with western blot data, where cell cycle promoting proteins cdc2 and its cyclin partners (cyclin A1 and cyclin B1) are down regulated post *R. stricta* treatment (Fig. 5f). Cdc2 activity is regulated through two mechanisms, first by binding to its cyclin partners [57], and second by being phosphorylated at Tyr15, and subsequently inhibited [58, 59]. Cdc2/cyclin complexes contribute to cell cycle progression through phosphorylation of target proteins [60]. As shown, Cdc2, and its partners cyclin A and cyclin B were downregulated in a time-dependent manner (Fig. 5f). In addition, treatment with *R. stricta* extract resulted in a slight increase in Tyr15 phosphorylated Cdc2 (Fig. 5f).

### *R. stricta* extract inhibited the colony formation of HepG2 cells

**Fig. 6:**
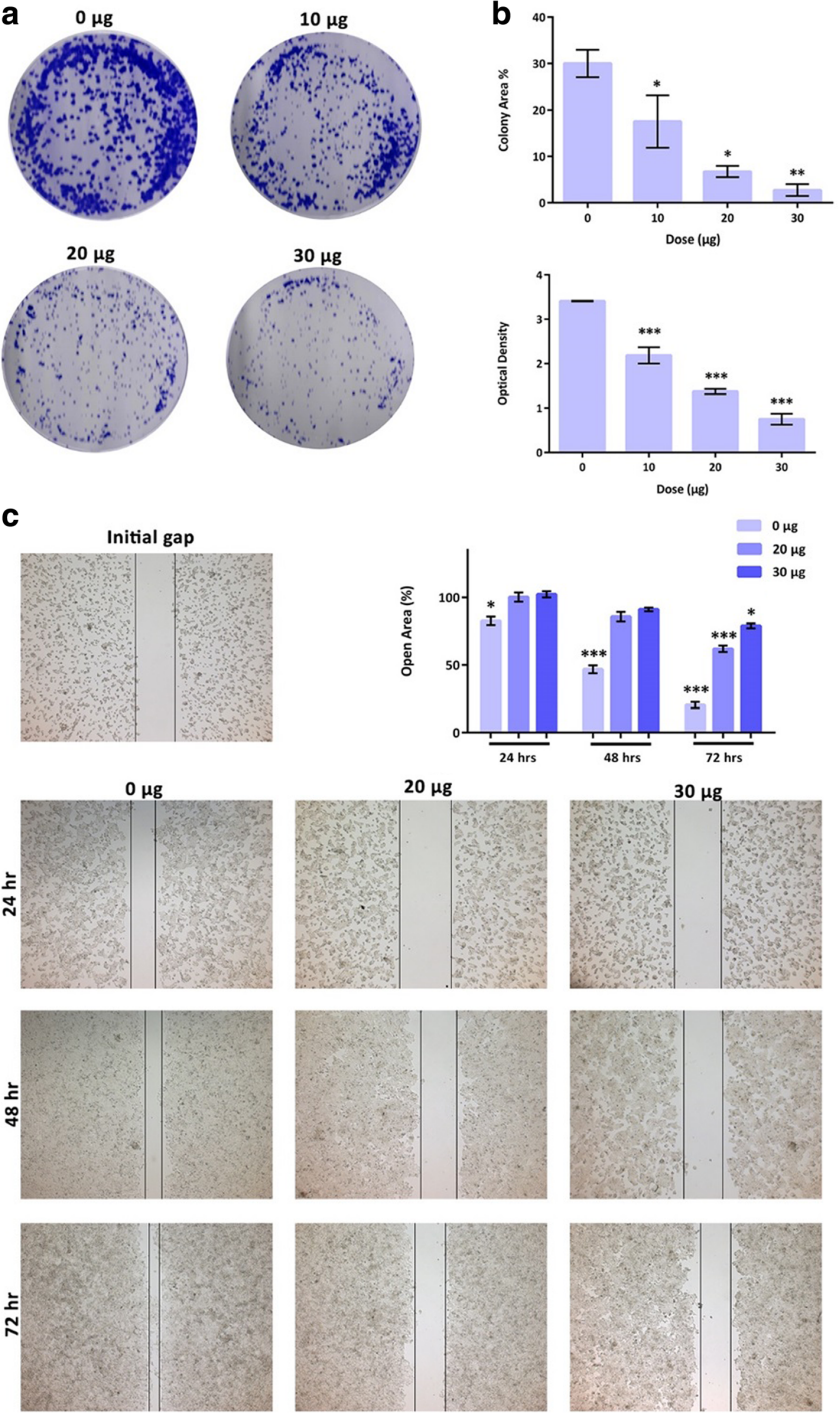
*Rhazya stricta* extract inhibits colony forming ability and wound healing of HepG2 cells in a dose-dependent manner. **a** Representative images of HepG2 colonies after treatment with increasing concentrations of *R. stricta* extract. **b** Percent of area occupied by colonies in treated and non-treated wells (representative of triplicate samples; *P < 0.05, **P < 0.005, ***P < 0.0001) and absorbance of each treated and non-treated wells (representative of 3 biological triplicates, each in triplicate; *P < 0.05, **P < 0.001, ***P < 0.0001). **c** Representative images and quantification (three regions of three biological triplicates; *P < 0.05, **P < 0.001, ***P < 0.0001) of wound-healing assay results of HepG2 cells treated without or with 20 and 30 μg of *R. stricta* extract

This assay measures the ability of tumour cells to survive and grow to form colonies after treatment with cytotoxic agents. To assess clonogenicity, cells were plated onto 6-well plates and incubated with 0.0, 10, 20, and 30 μg of *R. stricta* extract and treated cultures were maintained in culture for an additional 10 days to allow formation of colonies [61, 62]. The results from that assay showed that *R. stricta* extract inhibited the colony formation of HepG2 cells in a dose-dependent manner (0, 10, 20, 30 μg). ImageJ analysis results were consistent with results obtained from the absorption-based method where *R. stricta* extract also inhibited the survival of HepG2 cells in a dose-dependent manner (Fig. 6a and b).

### *R. stricta* extract inhibits the migration ability of HepG2 cells

Many cancer patients die of tumour mobilization which often lead to metastatic foci in distant part/s of the body. Therefore, attempts to prevent or slowdown progression and metastasis of cancer cells are crucial [63]. Earlier study showed that *R. stricta* extract exerts potent anti-cancer ability against human breast cancer [64]. To date, however, limited information is available regarding *R. stricta* extract effect on cell migration in any cancer type. The potential role of *R. stricta* extract on HepG2 cells’ migration was assessed using wound healing assay. Cell migration was inhibited in a dose-dependent manner (0, 20 and 30 μg) starting from 24 to 72 h post *R. stricta* extract treatments compared to the control (Fig. 6c).

### *R. stricta* extract inhibits endothelial cell tube formation

**Fig. 7:**
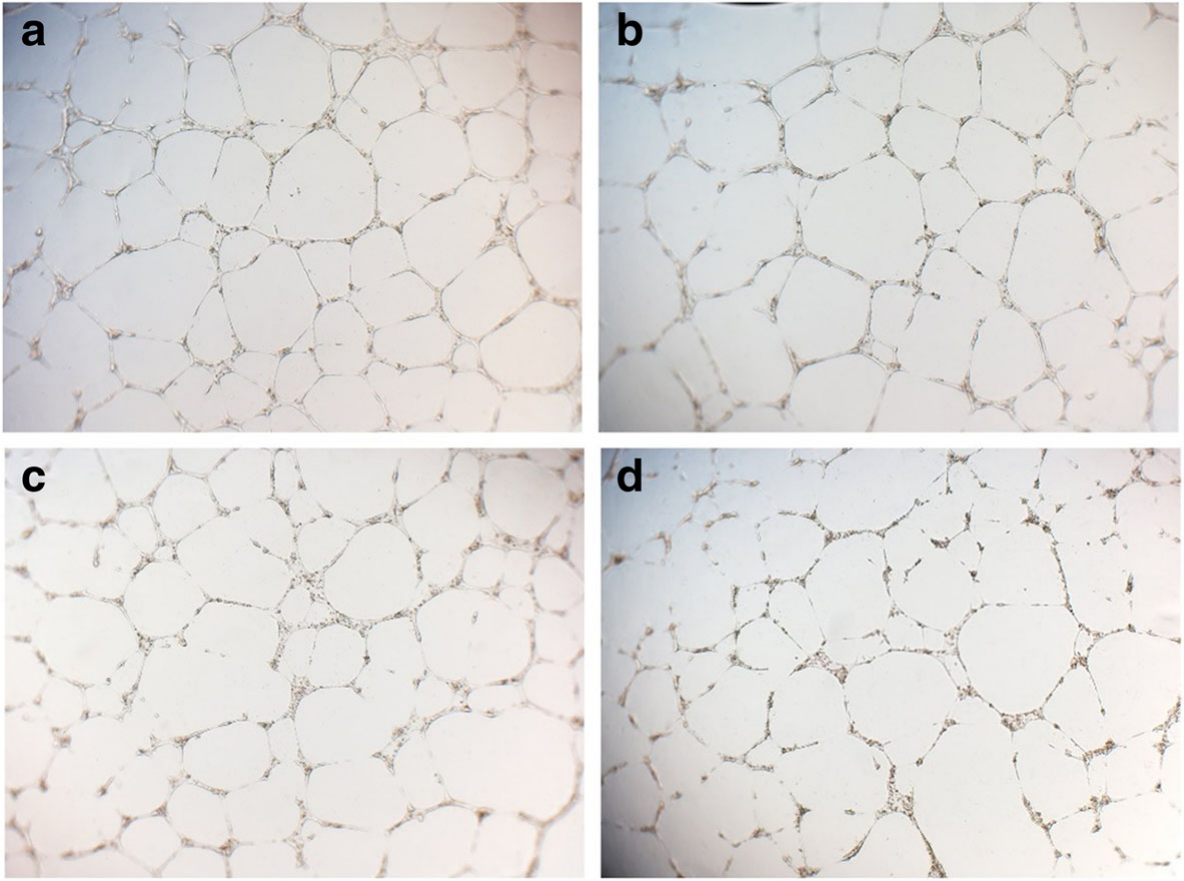
*Rhazya stricta* extract inhibits tube formation in HUVECs on Matrigel in a dose-dependent manner. Photographs of tube formation in HUVECs on Matrigel after incubation with or without *R. stricta* extract at 18 h. Cells were treated with *R. stricta* extract at a series of concentrations (10 μg; **b**, 20 μg; **c**, 30 μg; **d**) or DMSO vehicle (control; **a**) for 18 h

Cell migration and invasion are crucial steps in various physiological processes such as morphogenesis, angiogenesis, wound healing and inflammation [65]. Invasion and migration are critical events for tumour progression and tumour recurrence [66, 67]. HUVEC cells had greater numbers of branching tube networks after 18 h of incubation with no *R. stricta* extract (Fig. 7a). This tube branching was attenuated by *R. stricta* extract treatments in a dose-dependent manner (Fig. 7b and c). Colony formation results demonstrate that colony numbers in all dose groups were decreased. These results suggest that *R. stricta* extract reduced cell migration and colony formation capacity in HepG2 cancer cells. *R. stricta* extract may, therefore, be introduced as a novel agent to treat/prevent HCC and as a single agent or in combination with other drugs. It would also be of interest to identify *R. stricta* extract bioactive molecules and assess their effects against cancer, particularly HCC. Further studies are currently underway to identify and characterize *R. stricta* extract bioactive ingredients and to unravel their molecular mechanism against cancer.

The obtained antiproliferative results of the three extracts against HepG2 cancer cell line were found to be consistent with their antioxidant activity, free radical scavenging ability and phenolic content. Out of the three extracts, *R. stricta* showed considerable antiprolifrative activity at low concentration and was the most potent among the tested plants with IC_50_ = 30 μg/mL. In addition, *R. stricta* showed the highest total polyphenols (11.5 ± 0.013 mg GAE/g extract). It has also the most percentage inhibitions of DPPH scavenging activity (89.9% ± 0.51) at 1.5 mg/ml and (28.7% ± 1.27) at 0.15 mg/ml. This indicates that the antiproliferative effect of the three extracts, in particular *R. stricta*, may be attributed to their antioxidant polyphenolic and flavonoid contents. Phenolic compounds have been found to counteract cancer either by means of antioxidant effect or by inhibiting the formation of carcinogenic metabolites that damage the vital biomolecules [68].

Finally, the investigated UAE plants have shown a strong reducing antioxidant capacity and free radical scavenging ability. These findings validate the use of these plants in folk medicine for the treatment of certain diseases. Oxidative cell damage events are frequently correlated with the oxidative stress. We show here that both of those properties are present in the ethanolic extract of *R. stricta*, which has significant antiproliferative activity and a great antioxidant activity. The results reported here are very promising indicators for the potential application of this plant for preventive and therapeutic purposes.

## Conclusions

In conclusion, natural antioxidants have many important applications in health promotion, food preservation, food flavouring and cosmetics. They are preferred over synthetic antioxidants because they are safer for consumption and more environmentally friendly. The present study investigates the antioxidant activity and polyphenolic content of three medicinal plants from the UAE. We found extracts rich in antioxidants and in polyphenols, which merit further investigations. Of the extracts evaluated, that of *R. stricta* (Harmal) showed the greatest antioxidant, antiangiogenic and antiproliferative activities, a discovery that makes this species a promising source of anticancer agent development especially for colon and liver cancers, and hence worthy of further investigation. Isolation of active compounds and exploring their mode of action against tumours by using in vivo experimental models would make an important future study.

## Additional file

Additional file 1: **Figure S1.** Assessment of the cytotoxic effects of *Trigonella foenum-graecum* (Helba), *Cassia acutifolia* (Holoul), and *Rhazya stricta* (Harmal) extracts on HCT116 in vitro. MTT assay results of 9cHCT116 cells viability after treatment with increasing concentrations of Helba (a) Holoul (b) and Harmal (c) for 24 h. *P < 0.05, **P < 0.005, ***P < 0.0001. (DOCX 99 kb)
