# Supplementary material for: Antioxidant and anticancer activities of Trigonella foenum-graecum, Cassia acutifolia and Rhazya stricta
Source: BMC Complement Altern Med. 2018 Aug 22;18:240. doi: 10.1186/s12906-018-2285-7 (PMC6103858; doi:10.1186/s12906-018-2285-7)
Supplement: Supplementary file 1 — Figure S1. Assessment of the cytotoxic effects of Trigonella foenum-graecum (Helba), Cassia acutifolia (Holoul), and Rhazya stricta (Harmal) extracts on HCT116 in vitro. MTT assay results of 9cHCT116 cells viability after treatment with increasing concentrations of Helba (a) Holoul (b) and Harmal (c) for 24 h. *P < 0.05, **P < 0.005, ***P < 0.0001. (DOCX 99 kb) [file 12906_2018_2285_MOESM1_ESM.docx]

***Additional file***

**Antioxidant and Anticancer Activities of *Trigonella foenum-graecum,***

***Cassia acutifolia and Rhazya stricta***

**Bayan Al-Dabbagh^1,*^, Ismail A. Elhaty^1^, Ala’a Al Hrout^2^, Reem Al Sakkaf^1^, Raafat El-Awady^3^,**

**S. Salman Ashraf^1^, Amr Amin^2*^**

**^1^Department of Chemistry, College of Science, UAE University, Al Ain, PO Box 15551, UAE**

**^2^Department of Biology, College of Science, UAE University, Al Ain, PO Box 15551, UAE and Zoology Department, Cairo University, Giza, Egypt**

**^3^Department of Pharmacy Practice and Pharmacotherapeutics, Sharjah Institute for Medical Research and College of Pharmacy, University of Sharjah, Sharjah, UAE**

**Figure S1**


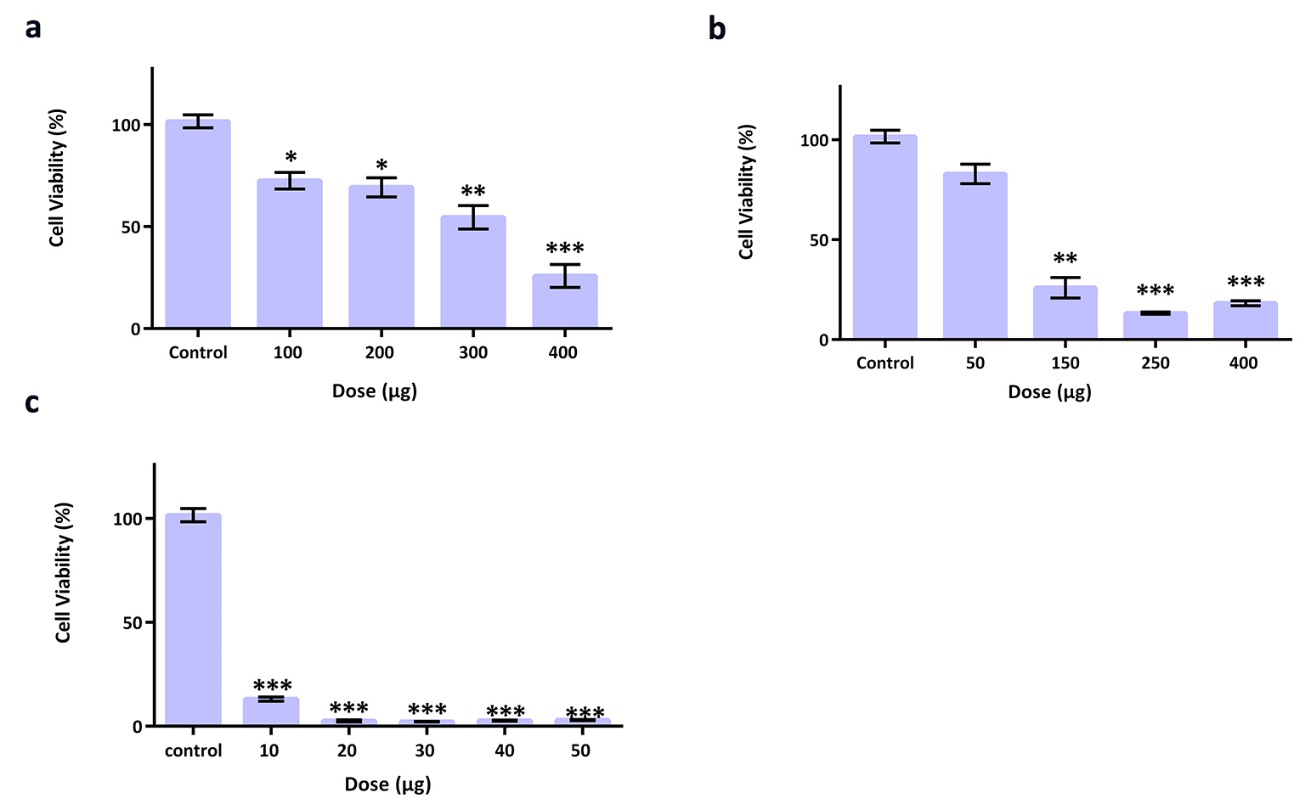


**Figure S1.** Assessment of the cytotoxic effects of *Trigonella foenum-graecum* (Helba), *Cassia acutifolia* (Holoul), and *Rhazya stricta* (Harmal) extracts on HCT116 *in vitro*. MTT assay results of 9cHCT116 cells viability after treatment with increasing concentrations of Helba (a) Holoul (b) and Harmal (c) for 24 hours. *P<0.05, **P<0.005, ***P<0.0001.
